# Calcium signaling in soybean-virus interactions: emerging roles of calcium sensors in antiviral and environmental stress responses

**DOI:** 10.3389/fpls.2026.1834840

**Published:** 2026-06-30

**Authors:** Huong Cam Chu, Jisuk Yu, Kook-Hyung Kim

**Affiliations:** 1 Department of Agricultural Biotechnology, College of Agriculture and Life Sciences, Seoul National University, Seoul, Republic of Korea; 2 Plant Genomics and Breeding Institute, Seoul National University, Seoul, Republic of Korea; 3 Research Institute of Agriculture and Life Sciences, Seoul National University, Seoul, Republic of Korea; 4 SNU Plant Health Center, Seoul National University, Seoul, Republic of Korea

**Keywords:** calcium signaling, resistance, soybean, soybean mosaic virus, virus-host interaction

## Abstract

Soybeans (*Glycine max*) in the field face biotrophic and abiotic stresses simultaneously. Although calcium (Ca^2+^) signaling mediates plant stress adaptation and links perception to downstream transcriptional and metabolic reprogramming, its roles in integrating viral defense in soybean remain under-investigated. Consequently, most soybean antiviral research has focused on *R* genes and RNA silencing rather than on the calcium signaling hubs that may coordinate and fine-tune these defense pathways. Here, we propose a calcium-centered framework for understanding soybean-virus interactions, with an emphasis on the soybean mosaic virus (SMV). We summarize the soybean calcium signaling modules including calcineurin B-like protein (CBL)-CBL-interacting protein kinase, calcium-dependent protein kinases, and calmodulins/calmodulin-like proteins, which are key regulators of abiotic stress tolerance in soybean; however, their direct roles in antiviral defense remain largely unexplored. Recent transcriptomic and weighted gene co-expression network analysis studies have identified Ca^2+^-linked nodes embedded in reactive oxygen species, salicylic acid/jasmonic acid and mitogen-activated protein kinase networks, which are likely to modulate basal and *R* gene-mediated resistance. Insights from other plant-virus systems further show that how viruses have evolved sophisticated counter-strategies to subvert these Ca^2+^-mediated host defenses. Emerging models in *Nicotiana benthamiana* and *Arabidopsis* demonstrate how viral proteins manipulate calcium hubs to suppress oxidative bursts and RNA interference machinery. Despite the challenges posed by the paleopolyploid nature of the soybean genome and the technical hurdles of function validation, this review emphasizes that deciphering the calcium-dependent pathways in the soybean-SMV pathosystem is essential for engineering broad-spectrum, durable virus resistance and ultimately contributing to sustainable crop protection.

## 1 Introduction

Soybeans (*Glycine max* (L.) Merr.) are a versatile crop essential for livestock feed, human health, and industrial applications, as well as renewable energy (Guo et al., 2022; Nikolić et al., 2025). Soybean fields are under attack from viral complexes (e.g., soybean mosaic virus, soybean yellow mottle mosaic virus, and bean common mosaic virus), non-viral pathogens (e.g., oomycetes, such as *Phytophthora sojae* and *Phytophthora* spp., and fungi, such as *Diaporthe* spp.), and abiotic stressors (e.g., drought, salinity, flooding, and temperature extremes). These factors trigger synergistic effects that significantly amplify disease severity (Whitham et al., 2016; Tripathi et al., 2022; Hosseini et al., 2023). These biotic pressures are further compounded by abiotic stressors, such as drought, salinity, flooding, and temperature extremes, which impair defense signaling and favor vector proliferation in humid environments (Suzuki et al., 2014; Liu et al., 2024a; Fang et al., 2025). Consequently, maintaining consistent soybean productivity and quality has become increasingly difficult. Therefore, understanding how soybeans respond to these multifactorial stresses is crucial for developing resilient cultivars.

Although calcium-mediated signaling is a well-established pathway under various abiotic and biotic stress conditions in plants (Jiang and Ding, 2023; Wang and Luan, 2024), its role in soybean has been studied primarily focused on the responses to abiotic stress adaptation (Ramlal et al., 2024; Fang et al., 2025). Consequently, the regulatory mechanisms by which calcium sensors integrate viral defense signals into broader physiological adaptations require extensive investigation. Emerging evidence suggests that calcium signaling may function as a pivotal molecular hub, integrating diverse defense layers including *R-*gene-mediated immunity, RNA interference (RNAi), and phytohormone signaling pathways (Jiang and Ding, 2023; Ali et al., 2024; Weralupitiya et al., 2024). Calcium ions play a decisive role in orchestrating the plant’s adaptive responses to multifactorial challenges by bridging the gap between environmental stress perception and complex biotic defense networks.

Plant viruses have been observed to manipulate calcium signaling hubs, either directly or indirectly, to subvert host defenses (Wang et al., 2021; Zvereva et al., 2024). Although soybean-virus interactions have been extensively studied in the context of *R* genes (Tripathi et al., 2022), the subsequent downstream signaling events and the manner in which viruses interfere with these calcium-mediated defense cascades remain poorly understood.

Soybean mosaic virus (SMV; genus *Potyvirus*), a major seed- and aphid-transmitted pathogen with diverse regional strains (G1–G7 and variants). The soybean-SMV pathosystem is a powerful model for studying plant-virus immunity and stress adaptation because of the well-characterized genetic architectures of the two organisms (Zhao et al., 2025). Of the more than 14 viruses identified in Korean soybean fields, SMV is the most prevalent and economically damaging pathogen, necessitating urgent strategies for both early detection and the development of robust defense mechanisms (Jo et al., 2020; Oh et al., 2024). Understanding calcium-dependent pathways with *R*-gene-mediated immunity provides a more comprehensive view of how soybeans coordinate systemic resistance to counteract the evolutionary adaptability of SMV. Therefore, this mini-review aims to synthesize recent findings and highlight the multifaceted roles of calcium signaling in soybeans, with a focus on its potential to interact with viruses and soybean defense through the downstream calcium signaling pathway.

## 2 Calcium signaling modules in soybean

### 2.1 Calcium sensors and kinase modules in soybean

Plants have a variety of calcium sensors that interpret transient calcium fluctuations. These sensors include calmodulins (CaMs), calmodulin-like proteins (CMLs), calcium-dependent protein kinases (CDPKs), and calcineurin B-like proteins (CBLs). These sensors typically harbor EF-hand motifs that undergo conformational changes upon Ca²^+^ binding. This modulates their enzymatic activities or interactions with downstream targets (Zeng et al., 2023). CDPKs are unique in that they act as both sensors and responders through integrated kinase and Ca^2+^ binding domains (Deng et al., 2025; Azupio et al., 2026). Conversely, CaMs and CBLs lack intrinsic catalytic activity and depend on interacting partners, such as CaM-kinases (CamKs) or CBL-interacting protein kinases (CIPKs), to transduce Ca^2+^ signals (Luan and Wang, 2021). CaMs are evolutionarily conserved across eukaryotes, but the diversification of CMLs, CBLs, and CDPKs is plant-specific. The structures, modes of action, and contributions of each class of Ca^2+^ sensor to plant stress responses have been thoroughly reviewed (Zeng et al., 2023; Bihani et al., 2025; Ren et al., 2025). This expanded signaling complexity likely reflects an adaptation of sessile organisms to survive and navigate fluctuating environmental conditions (Edel et al., 2017).

In soybean, genome-wide analyses have identified various calcium signaling components, thereby expanding our understanding of its intricate regulatory networks. Comprehensive gene families have been characterized to date, including calcium transporters (Zeng et al., 2020; Jia et al., 2022), *GmCDPKs* (Liu et al., 2016)*, GmCAMs* (Zhang et al., 2025), *GmCMLs* (Yadav et al., 2022), *GmCBLs* (Jiao et al., 2024), and *GmCIPKs* (Zhu et al., 2016; Jiao et al., 2024). The total number of calcium sensors identified in soybeans and their representative members are summarized in the **Table 1**. This functional partitioning suggests that soybean calcium signaling should not viewed as a single pathway, but rather as a layered network in which distinct sensor families mediate different phases and intensities of stress adaptation.

**Table 1:** List of calcium sensors that have been identified in soybean (*Glycine max*).

Family	No. of genes in glycine max	Key structural features	Primary localization	Reference
Calcineurin-B like proteins (CBL)	15	EF-hands and N-terminal myristoylation site	Plasma membrane, Endomembranes	(Xu et al., 2021; Jiao et al., 2024)
CBL-interacting protein kinase	52	NAF/FISL domain (CBL binding)		(Zhu et al., 2016)
Calcium dependent protein kinases (CDPKs)	50^a^(39^b^)	EF-hand motifs and Kinase domain	Cytosol, Plasma membrane	(aHettenhausen et al., 2016; bLiu et al., 2016)
Calmodulin (CaMs)	11	Conserved EF-hands (Sensor only)	Nucleus, Cytosol	(Zhang et al., 2025)
Calmodulin-like proteins (CMLs)	102	Diverged EF-hands (Sensor only)	Nucleus, Cytosol	(Yadav et al., 2025; Zhang et al., 2025)
MCUs	11(Glycine soja)	Conserved MCU domain (PF04678)	Mitochondrial membrane	(Li et al., 2022c)
P2-type ATPases	33 (GmACAs, GmECAs)	Autoinhibitory domain and DKTGT, PEGL, and KGAXE motifs	Plasma membrane, Tonoplast	(Zhao et al., 2020)

^a^ Identified 50 gene loci (63 isoforms) by screening for kinase and EF-hand domains.

^b^ Identified 39 non-redundant genes by querying rice and *Arabidopsis* sequences and manually confirming the presence of kinase and calmodulin-like domains.

### 2.2 Calcium signaling in the abiotic stress response of soybean

Soybean is frequently exposed to various abiotic stresses, including drought, salinity, alkalinity, cold, flooding and shading, all of which impact growth and yield (Fang et al., 2025). Under these abiotic stress conditions, stress-induced Ca^2+^ signals are perceived by multiple classes of calcium sensors, including CaMs, CMLs, CDPKs/CPKs, and CBLs, as well as organelle-associated calcium transport systems such as Ca^2+^-ATPase and mitochondrial calcium uniporter-related components (Leung et al., 2023; Rui et al., 2025). These signaling modules translate transient calcium elevations into physiological responses by regulating reactive oxygen species (ROS) homeostasis, abscisic acid (ABA) signaling, stomatal movement, ion balance, MAPK cascades, and stress-responsive transcription factors (Fang et al., 2025; Azupio et al., 2026).

The *GmCBL*-*GmCIPK* network is well characterized for its role in abiotic stress with its structural diversity and the presence of various hormone-responsive *cis*-acting elements (Chen et al., 2024; Jiao et al., 2024). Analysis of *GmCBL* promoters reveals a wide spectrum of regulatory motifs, indicating that these modules serve as critical integration points for external environmental cues and internal defense signaling (Jiao et al., 2024). Under salt and alkaline stress, specific modules, such as GmCBL1- GmCIPK, GmCBL4-GmCIPK2 and GmCBL4-GmCIPK21, function as regulators, modulating redox homeostasis, enhancing antioxidant enzyme activities, and maintaining the balance of Na^+^/K^+^ ions (Li et al., 2022a, 2022b). Meanwhile, the GmCBL9-GmCIPK6 complex improves salt tolerance by facilitating K^+^ influx mediated by GmAKT1 through the phosphorylation of K^+^ channels (Feng et al., 2025). In terms of drought resistance and ABA signaling, GmCIPK2 and GmCIPK29 act as positive regulators. By interacting with GmCBL1, these kinases enhance ABA sensitivity and mediate ROS scavenging under water deficit conditions (Xu et al., 2021; Wang et al., 2023). Together, these modules link the perception of environmental stress with the activation of defense mechanisms, including ROS scavenging systems and ion transporters. Previous studies have shown that the majority of GmCDPKs respond to drought or ABA treatments (Hettenhausen et al., 2016). More recently, *GmCDPK5* has been characterized as a salt- and drought-responsive gene that exhibits a more sensitive transcriptional response in wild soybean (*G. soja*) (Veremeichik et al., 2022).

The expression of mitochondrial calcium uniporters (MCUs) and P2-type Ca^2+^ ATPase genes (*GmACA*s and *GmECA*s) is differentially regulated under various abiotic stresses, including drought, salinity, and cold (Zhao et al., 2020; Li et al., 2022c; Wang et al., 2022b). Specifically, seven *GsoMCU* genes in *G. soja* exhibit significant transcriptional up-regulation in response to cold and alkaline stresses (Li et al., 2022c). Under cold stress, six up-regulated *GmACA* genes serve as potential positive regulators of cold-induced stomatal closure, whereas GmACA1 and GmECA3 appear to play negative roles (Wang et al., 2022b). These findings suggest that a complex, fine-tuned regulatory network comprising Ca^2+^ pumps and transport modules, contributes to soybean resilience against abiotic constraints. **Table 2** summarizes the calcium sensors identified in soybean in response to biotic and abiotic stresses.

**Table 2:** Comparative summary of calcium signaling pathways under biotic and abiotic stresses.

Stress category	Specific stress type	Calcium sensors	Key signaling mechanisms & responses	Reference
Abiotic Stress	Cold (Low Temp)	GsoMCUs, GmACAs, GmECAs	Mitochondrial Ca^2+^ uptake; Stomatal closure regulation	Li et al., 2022c; Wang et al., 2022b
	Drought & Salinity	GmACAs	ABA-dependent transcriptional regulation; Osmotic adjustment	Wang et al., 2022b
		GsoMCU2, 5, 6	Mitochondrial Ca^2+^ signaling for pH and ion homeostasis	Li et al., 2022c
		GmCBL1, 4	Promoted the expression of salt- and antioxidant-related genes, ABA-dependent transcriptional regulation, and ROS scavenging	Li et al., 2022a; Li et al., 2022b; Wang et al., 2023; Jiao et al., 2024
		CIPK2, 21, 29		
	Alkaline	GmCDPK5	ABA-dependent transcriptional induction	Veremeichik et al., 2022
		GmCBL9-GmCIPK6	Phosphorylate GmAKT1, ion transport regulation, restore Na+/K+ homeostasis	Feng et al., 2025
	Shading	GmECA1	Negative regulation of stomatal opening under low light	Wang et al., 2022b
Biotic Stress	Fungal and/or viral infection	GmCaM4, GmCaM5	Enhanced resistance via SA-mediated PR gene induction	Heo et al., 1999
		GmCDPKs	JA/SA-mediated PR gene induction; ROS-dependent defense	Rao et al., 2014
		GmCaMs, GmCML66	Transcriptional induction patterns highly correlated with virus resistance strain	Niu et al., 2024
		GmCML23, GmCML47, GmCaM4	Transcriptional induction patterns highly correlated with resistance phenotypes	Zhang et al., 2025
	Insect Herbivory	GmCML77	Ca^2+^ dependent conformational changes to mediate downstream signaling	Yadav et al., 2025
		GmCDPK17, GmCDPK38	Positively Regulates Soybean Resistance to common cutworm	Wang et al., 2022a

### 2.3 Potential roles of calcium sensors in soybean antiviral immunity

Ca^2+^ signaling has been well-established as a core module for abiotic stress adaptation in soybean, their involvement in virus-host interactions has not been sufficiently explored. CaMs and CDPKs integrate Ca^2+^ bursts into downstream mitogen-activated protein kinase (MAPK) pathways during stress responses (Liu et al., 2024b; Deng et al., 2025). In soybean-pathogen interactions, these sensors regulate pathogenesis-related (*PR*) gene induction, which offers a foundation for antiviral applications that remain to be explored. GmCaM4 enhances resistance against a broad spectrum of pathogens by inducing the accumulation of jasmonic acid (JA) and the expression of *PR* genes in response to pathogens (Rao et al., 2014). Additionally, overexpressing GmCaM4 or GmCaM5 in transgenic tobacco enhances resistance against pathogens, including the tobacco mosaic virus, by constitutively expressing defense-related genes via a salicylic acid (SA)-independent signaling pathway (Heo et al., 1999). These results suggest that specific GmCaM isoforms restrict viral accumulation via robust, independent signaling networks. Recent evidence in *Nicotiana benthamiana* links calcium signaling, triggered by wounding, directly to the RNAi machinery. There, a CaM-binding transcription activator-like protein 3 (CAMTA3) module activates the downstream components bifunctional nuclease-2 (BN2) and RNA-dependent RNA polymerase 6 (RDR6) (Wang et al., 2021). BN2 stabilizes the mRNA of core RNAi components, such as AGO1/2 and DCL1, by degrading their cognate microRNA. This process primes the cell for antiviral defense. Given the established roles of GmCaMs in soybean immunity, it is probable that a similar calcium-mediated RNAi antiviral defense response exists in soybean. GmCML77 has been shown to be a functional calcium sensor that undergoes significant conformational changes and hydrophobic residue exposure upon Ca^2+^ binding during insect infestation (Yadav et al., 2025).

Although CDPKs are well-established regulators of plant immunity against various pathogens and pests, their specific roles in soybean antiviral defense are less well understood than those in other model plants. In *Arabidopsis* and tobacco, certain CDPKs positively regulate broad-spectrum immunity by triggering SA accumulation and ROS bursts against fungal and bacterial pathogens. In contrast, their homologues in barley and tobacco act as negative regulators; silencing them leads to enhanced resistance through the accumulation of JA or secondary metabolites (Kiselev and Dubrovina, 2025). A recent study showed the diverse roles of the GmCDPK subfamily in soybean immunity. GmCDPK38 acts as a negative regulator against the common cutworm, while GmCDPK17 functions as a positive regulator and is a key player in insect resistance (Wang et al., 2022a).

The GmCBL-GmCIPK network is well characterized in the response to abiotic stress. However, its structural diversity and hormone-responsive *cis*-acting elements suggest an unexplored role in soybean-virus interactions (Chen et al., 2024; Jiao et al., 2024). Insights from model plants, such as *N. benthamiana*, provide compelling evidence of their involvement in antiviral immunity. Recent studies have revealed that NbCBL10 expression is induced by viral infections and that silencing this gene results in increased viral accumulation (He et al., 2025). These findings indicate a potential role for the CBL-CIPK module in soybean antiviral surveillance and suggest that this network may contribute to sustained defense and metabolomic stability (He et al., 2025).

## 3 Soybean-virus interactions revisited through a calcium lens

### 3.1 Transcriptomic insights into calcium signaling during SMV infection

To understand the role of calcium sensors in response to diverse abiotic and biotic stress factors, a comprehensive transcriptomic analysis was performed to identify dynamically reprogrammed calcium-related genes in soybean. Several studies have identified significant changes in Ca^2+^ transporter genes under salt stress, phosphorus deficiency, and fungal pathogen inoculation (Jia et al., 2022; Zhou et al., 2025a). Similarly, specific clusters of *GmCDPKs* and *GmCMLs* exhibit differential expression during wounding, herbivore attacks, and pathogen invasions (Hettenhausen et al., 2016; Liu et al., 2016; Zhang et al., 2025). Tracking the differential expression patterns of calcium signaling-related genes identifies key candidates that serve as pivotal regulatory nodes, bridging basal metabolism and active antiviral defense. However, direct functional validation in soybeans is an emerging field.

Recent analyses of resistant and susceptible soybean cultivars following SMV infection has revealed significant differences in gene expression. Key components of Ca^2+^ signaling, such as *GmCDPK, GmCaMs, GmCMLs*, and *GmCNGCs*, exhibited distinct expression patterns depending on the time post-infection and the cultivar (Li et al., 2023; Niu et al., 2024). These findings suggest that genes related to Ca^2+^ signaling play a crucial role in modulating dynamic early immune responses against viral infection. Furthermore, 15 out of 113 *GmCaM*/*CML* genes were found to be differentially expressed in response to SMV and the fungal pathogen *Cercospora sojina* (Zhang et al., 2025). Several of these genes showed correlations with resistance phenotypes, suggesting their potential roles in mediating broad-spectrum disease resistance in soybeans. Weighted gene co-expression network analysis (WGCNA) in soybean identified *GmCML66* (Glyma.12G1285400; (Li et al., 2026) suggested as a pivotal hub gene significantly associated with resistance to the SMV SC15 strain, showing higher expression in resistant lines compared to susceptible ones (Niu et al., 2024). This CML hub, along with other calcium-dependent regulators such as GmCDPK, is integrated into a multilayered signaling network involving Ca^2+^ ROS, and ABA pathways. This suggests that CML plays a critical role in orchestrating the early immune response and hormonal crosstalk in soybean (Niu et al., 2024). These altered expression profiles not only establish a molecular link between calcium flux and viral pathogenesis, as well as a regulatory hub associated with *R* gene-mediated or systemic resistance in soybean.

### 3.2 *R* gene clusters and Ca^2+^ crosstalk in broad-spectrum resistance

Recent studies have highlighted the integration of Ca^2+^ signaling within soybean *R* gene clusters, particularly by SRC4 (SMV Resistance Cluster), which is located on chromosome 16 (Yan et al., 2022). SRC4 exhibits a uniquely high basal expression pattern, describing a primed state for immediate defense. Unlike typical NBS-LRR resistance genes, SRC4 harbors a Ca^2+^-binding EF-hand domain that enables it to simultaneously mediate viral resistance and abiotic stress responses (Zhou et al., 2025b). This dual responsiveness is grounded in a Ca^2+^-SA hierarchy: Ca^2+^ influx, triggered by either pathogen recognition or temperature fluctuations, acts as an upstream signal for SA biosynthesis, which subsequently induces SRC4 transcription. The enhanced temperature tolerance observed in SRC4-overexpressing plants further identifies Ca^2+^ signaling as a common crosstalk node that integrates biotic and abiotic stress adaptations. These findings suggest that broad-spectrum resistance is not solely defined by the breadth of pathogen recognition but also reflects a deeply integrated stress-response architecture. Ultimately, SRC4 provides compelling molecular evidence that the convergence of Ca^2+^ signaling pathways enables plants to orchestrate an integrated defense strategy against diverse environmental challenges.

### 3.3 Manipulation of the calcium signaling pathway by viruses: Broader lessons from plant-virus interactions

Viruses can manipulate the host’s calcium-mediated surveillance system to facilitate replication and systemic movement. The review by Zvereva et al., (Zvereva et al., 2024) emphasized the role of calcium in plant avtiviral defense from the pathogen's perspective. The review detailed the diverse strategies viruses use to hijack these pathways and enhance virulence. However, direct evidence of calcium signaling modulation in soybean during viral infection remains elusive. Nevertheless, models established in other plant-virus pathosystems provide a framework for potential regulatory interactions in soybean.

For example, the Ca^2+^-sensing module, which involves CaM and CAMTA3 and exhibits robust RNAi-mediated defense, is targeted by viral suppressors such as the geminivirus V2 protein or the wheat yellow mosaic virus P1 protein, which subvert host immunity (Wang et al., 2021; Chen et al., 2023). Similarly, Ca^2+^ sensors, such as CML and CPKs, play a crucial role in activating the oxidative burst and MAPK signaling pathways, thereby restricting viral accumulation. Tobacco mosaic virus (TMV) hijacks IP-L to degrade NbCML30 (Liu et al., 2022). Reduced NbCML30 abundance enables TMV to suppress the Ca^2+^-activated oxidative burst and reprogram resistance-related pathways. A recent study showed that Plantago asiatica mosaic virus infection triggers a systemic Ca^2+^ wave and subsequent CPK3-RBOHD-mediated ROS accumulation in cells surrounding infection foci. This restricts viral spread in *A. thaliana* (Ahmed et al., 2026). Conversely, the virus actively counteracts this defense by transcriptionally downregulating key components, including RBOHD and CNGC19, within infection foci. These results suggest that the virus selectively suppresses the host’s Ca^2+^ and ROS machinery to create a favorable, low-oxidative environment locally that facilitates replication and intercellular movement (Ahmed et al., 2026).

Chloroplasts also function as important hubs for calcium signaling during plant immunity. Specialized sensors in these hubs integrate signals from light, abiotic stress, and defense pathways (Nomura et al., 2012; Zvereva et al., 2024). Viral proteins, such as tomato yellow leaf curl virus C4, can relocalize from the plasma membrane to the chloroplast, where they interact with a calcium-sensing protein (CAS). This interaction inhibits SA biosynthesis and cytosolic Ca^2+^ bursts (Medina-Puche et al., 2020). Several soybean chloroplast-localized proteins, such as PsaC, ATPsyn-α, and GmMMP, have been reported to interact with various SMV proteins to modulate viral replication and ROS production (Bwalya et al., 2023; Zhou et al., 2026). It has been hypothesized that soybean-infecting begomoviruses or other ssDNA/RNA viruses may employ similar counter-defense strategies to manipulate chloroplast-mediated immunity through Ca^2+^ manipulation.

## 4 Future perspectives

**Figure 1:**
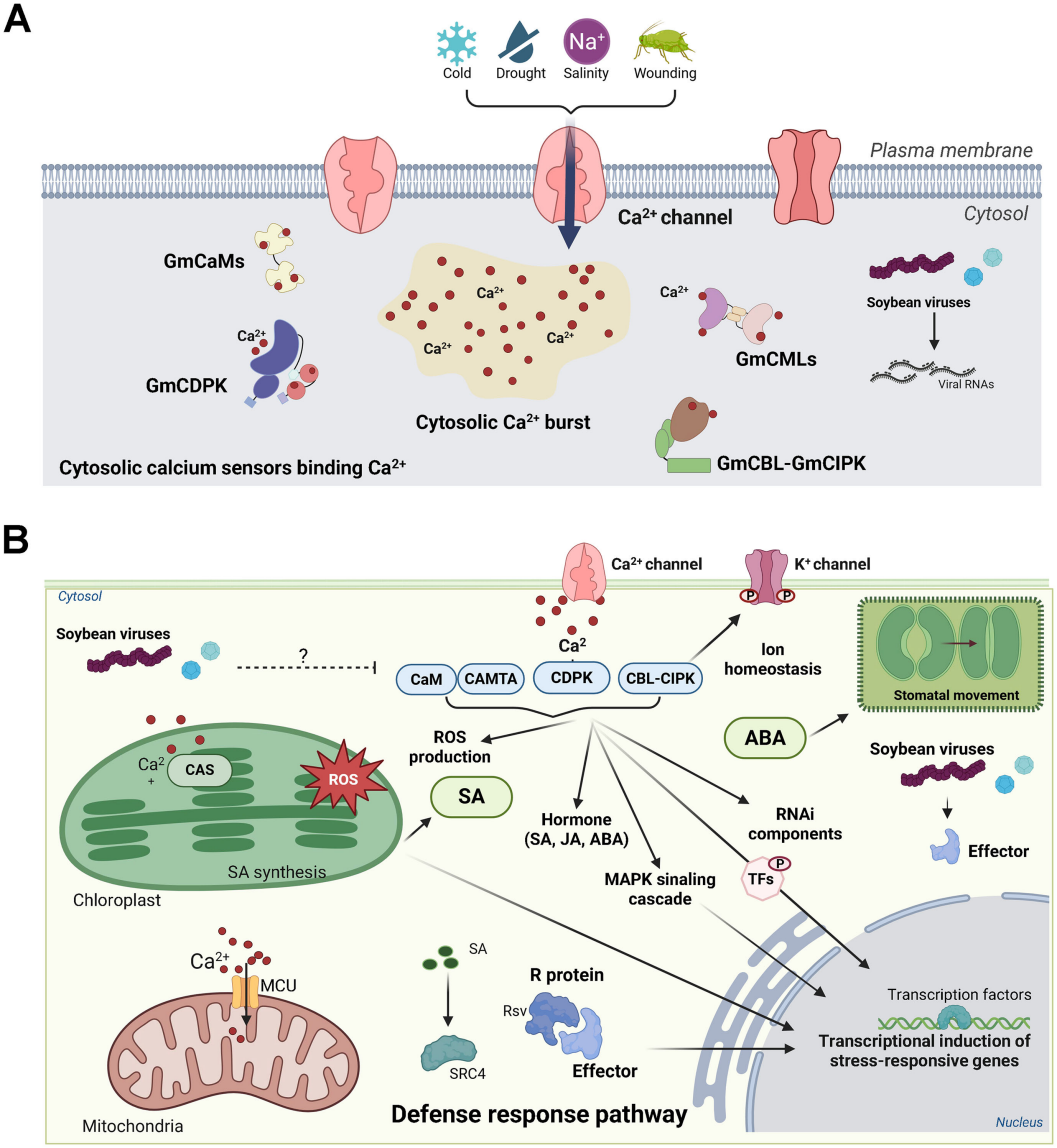
Conceptual model of calcium-mediated antiviral immunity in soybeans. **(A)** Abiotic and biotic stressors induce calcium signaling. Viral entry triggers a calcium flux that initiates viral replication and protein synthesis within the host cell. **(B)** Molecular regulatory networks mediated by calcium sensor proteins in soybean defense responses. Ca^2+^ sensors, including CaM, CBL-CIPK, and CDPK, coordinate the activation of reactive oxygen species (ROS) bursts and hormone-dependent defense pathways. Specific transcription factors (TFs) are phosphorylated and activated to modulate the expression of downstream defense genes. Calcium sensors localized in the chloroplast and mitochondria also contribute to the systemic defense machinery. Salicylic acid (SA) signaling induces the soybean mosaic virus resistance cluster 4 (SRC4) resistant protein, which confers broad-spectrum resistance. Viral effector proteins (e.g., SMV P3, HC-Pro, and CI) interact with the Rsv1, 3, and 4 resistant proteins to elicit resistance responses. Inhibitory T-bars indicate possible strategic points at which viral proteins interfere with these host surveillance pathways to facilitate infection. These illustrations were created with BioRender (BioRender.com).

This review synthesizes the current knowledge and recent findings on calcium signaling-mediated resistance, with a specific focus on the interaction between soybeans and viruses. **Figure 1** illustrates the proposed mechanisms by which soybean calcium signaling components orchestrate antiviral defense. This schematic shows how diverse calcium sensors act as a central hub to detect viral intrusion and activate downstream immune responses, including the *R* gene-mediated antiviral response pathway. These findings underscore the importance of the seamless integration of multiple signaling pathways for sustainable plant defense, with Ca^2+^ signals serving as the central convergence hub. Moving forward, leveraging genome-wide identification and comprehensive omics data will be important. Future research should prioritize identifying the direct downstream targets of Ca^2+^-related genes and clarifying the precise molecular mechanisms by which calcium sensor proteins confer robust resistance. However, significant challenges remain. The paleopolyploid nature of the soybean genome, which includes extensive gene duplication, creates functional redundancy that masks mutant phenotypes and complicates the functional assignment of highly conserved paralogs (Schlueter et al., 2007; Schmutz et al., 2010). Additionally, the low efficiency of stable transformation and the limitations of transient systems, such as the variability of virus-induced gene silencing and tissue-specific hairy root assays, hinder comprehensive functional validation. Overcoming these methodological hurdles requires a multifaceted strategy. CRISPR-based multiplex genome editing should be employed to disrupt paralogous Ca^2+^ signaling genes simultaneously to bypass redundancy. Specifically, leveraging advanced CRISPR toolkits, including Cas12a for multi-gene targeting and base/prime editors for precise nucleotide substitutions, is essential to overcoming the limitations of functional redundancy and broadening the scope of trait improvement in soybeans (Freitas-Alves et al., 2025). Integrating phosphoproteomics with coexpression network analysis will enable identifying the specific substrates of Ca^2+^ sensors during viral infection. Quantifying downstream signaling outputs and validating causal relationships in stable mutant backgrounds will allow us to move beyond single-gene studies and reveal the combinatorial logic of soybean calcium signals during stress and development. Closing the knowledge gap between calcium sensing and the activation of terminal defense effectors is essential for developing soybean varieties that are more climate-resilient and capable of withstanding simultaneous abiotic and biotic stresses.
